# Carbapenem-Resistant *Citrobacter* spp. as an Emerging Concern in the Hospital-Setting: Results From a Genome-Based Regional Surveillance Study

**DOI:** 10.3389/fcimb.2021.744431

**Published:** 2021-11-11

**Authors:** Yancheng Yao, Linda Falgenhauer, Jane Falgenhauer, Anja M. Hauri, Petra Heinmüller, Eugen Domann, Trinad Chakraborty, Can Imirzalioglu

**Affiliations:** ^1^ Institute of Medical Microbiology, Justus Liebig University Giessen, Giessen, Germany; ^2^ German Center for Infection Research (DZIF), Partner Site Giessen-Marburg-Langen, Justus-Liebig University Giessen, Giessen, Germany; ^3^ Institute for Hygiene and Environmental Medicine, Justus Liebig University Giessen, Giessen, Germany; ^4^ Department of Epimeiology, Hessisches Landesprüfungs- und Untersuchungsamt im Gesundheitswesen (HLPUG), Dillenburg, Germany; ^5^ Department of Epimeiology, Helmholtz Centre for Infection Research, Braunschweig, Germany

**Keywords:** *Citrobacter*, Carbapenemase, Germany, WGS, IncN-plasmid, MLST, ARGs

## Abstract

The rise of Carbapenem-resistant Enterobacterales (CRE) represents an increasing threat to patient safety and healthcare systems worldwide. *Citrobacter* spp., long considered not to be a classical nosocomial pathogen, in contrast to *Klebsiella pneumoniae* and *Escherichia coli*, is fast gaining importance as a clinical multidrug-resistant pathogen. We analyzed the genomes of 512 isolates of 21 CRE species obtained from 61 hospitals within a three-year-period and found that *Citrobacter* spp. (*C. freundii, C. portucalensis, C. europaeus, C. koseri and C. braakii)* were increasingly detected (n=56) within the study period. The carbapenemase-groups detected in *Citrobacter* spp. were KPC, OXA-48/-like and MBL (VIM, NDM) accounting for 42%, 31% and 27% respectively, which is comparable to those of *K. pneumoniae* in the same study. They accounted for 10%, 17% and 14% of all carbapenemase-producing CRE detected in 2017, 2018 and 2019, respectively. The carbapenemase genes were almost exclusively located on plasmids. The high genomic diversity of *C. freundii* is represented by 22 ST-types. KPC-2 was the predominantly detected carbapenemase (n=19) and was located in 95% of cases on a highly-conserved multiple-drug-resistance-gene-carrying pMLST15 IncN plasmid. KPC-3 was rarely detected and was confined to a clonal outbreak of *C. freundii* ST18. OXA-48 carbapenemases were located on plasmids of the IncL/M (pOXA-48) type. About 50% of VIM-1 was located on different IncN plasmids (pMLST7, pMLST5). These results underline the increasing importance of the *Citrobacter* species as emerging carriers of carbapenemases and therefore as potential disseminators of Carbapenem- and multidrug-resistance in the hospital setting.

## Introduction

The increase in infections caused by carbapenem-resistant Enterobacterales (CRE) represents a worrying threat to patient safety and healthcare systems worldwide and patients with CRE infections oftentimes cannot be treated effectively with available antibiotics (Grundmann et al., 2017). In contrast to Carbapenem-resistant *Klebsiella pneumoniae* and other carbapenem-resistant species, such as *Escherichia coli* and *Enterobacter* spp. which are commonly detected in nosocomial settings (Brolund et al., 2019; Pitout et al., 2020), *Citrobacter* was not considered to be a classical nosocomial pathogen. *Citrobacter* species belong to a group of facultative, anaerobic, Gram-negative bacilli within the *Enterobacteriaceae* family. They are frequently found in water, soil, food, and intestines of animals and humans and are mostly recognized as environmental contaminants or harmless colonizers. However, outbreaks in hospital settings are described, mostly occurring in neonates, young children or immunocompromised patients (Lipsky et al., 1980; Liu et al., 2017). They are able to cause a wide spectrum of infections involving the urinary tract, liver, biliary tract, peritoneum, intestines, bone, respiratory tract, endocardium, meninges, and the bloodstream. In addition, *Citrobacter* spp. are now fast gaining importance as a clinical multidrug-resistant pathogen causing opportunistic nosocomial and community-acquired infections (Mohanty et al., 2007; Villa et al., 2013; Wang et al., 2015; Arana et al., 2017a; Oyeka and Antony, 2017; Yao et al., 2017; Babiker et al., 2020). The carriage of antibiotic-resistant *C. freundii* was a mortality risk factor for immunocompromised patients with bacteremia (Liu et al., 2018). Recruitment of mobile colistin resistance, e.g. of the *mcr-1* gene, could further limit antibiotic therapeutic options (Hu et al., 2017; Giani et al., 2018).

In a regionally performed three-year-CRE-genomic surveillance study in the state of Hesse, Germany, 512 CRE isolates from 21 species, of which 368 were carbapenemase-producing Enterobacterales (CPE) underwent whole-genome analysis. Here we could demonstrate that the carbapenemase-gene-harbouring *Citrobacter* spp. contributed a high proportion among the CPE and the carbapenemase-producing *Citrobacter freundii* (CPC) was the third most frequently (~14%) identified species, following *Klebsiella pneumoniae* and *Escherichia coli*. CPC has increased in both absolute abundance and proportion in 2018 and 2019 compared to 2017, representing an emerging infection control and public health challenge (Yao et al. unpublished data). In the present study, the characteristics of carbapenem-resistant *Citrobacter* spp. are discussed in detail based on the results of the performed genomic analysis.

## Materials and Methods

### Bacterial Isolates

CRE isolates, including *Citrobacter* spp. isolates were obtained as part of the project of genome-based epidemiological surveillance study of carbapenem-resistant Gram-negative bacteria (CRGNB) in Hesse in Germany (SurvCARE Hessen). During the study period, 61 hospitals voluntarily participated. CRGNB definition followed guidelines of the Reporting Obligation Regulation Amendment by the Robert-Koch-Institute (RKI), the German national public health insitute (Robert Koch-Institute, 2016). Detected CRGNB must be reported to public health authorities. Mandatorily reported CRGNB isolates were included in the study.

### Bacterial Species Identification and Antimicrobial Susceptibility Testing

Antimicrobial susceptibility results obtained in the respective hospitals were confirmed centrally using the VITEK^®^ 2 system (bioMérieux, Nürtingen, Germany) and interpreted following EUCAST guidelines. Taxonomy was confirmed using MALDI-TOF-MS (Vitek MS, bioMérieux, Nürtingen, Germany).

### Bacterial Whole-Genome Sequencing

#### Short-Read Sequencing

For isolates that were non-susceptible per EUCAST definition to at least one carbapenem, short-read whole-genome sequencing, post-sequencing quality control and assembly were performed as described previously (Falgenhauer et al., 2020), and if needed, CLC Genomics Workbench v.10.1.0 (Qiagen, Aarhus, Denmark) also used. The average read length was 127 nt, the average coverage 93x.

#### Long-Read Sequencing

Two short-read sequenced *C. freundii* isolates were chosen for long-read sequencing in order to study carbapenemase-harbouring plasmids in more depth. DNA extraction and purification as described earlier (Falgenhauer et al., 2020). Hybrid assembly of short and long reads was performed using Unicycler implemented in the ASA^3^P pipeline (Schwengers et al., 2019).

### Analysis of Genomes and Plasmids

Identification of the chromosomal Multi-Locus sequence types (MLST), plasmid incompatibility (Inc) groups, plasmid MLST (pMLST) as well as acquired antibiotic resistance genes was performed using the Center for Genomic Epidemiology platform (https://cge.cbs.dtu.dk/services/) and the PubMLST database (https://pubmlst.org; https://bigsdb.pasteur.fr/cgi-bin/bigda.pl?db.). Species identification was performed using TYGS (https://tygs.dsmz.de/) (Meier-Kolthoff and Göker, 2019).

Single Nucleotide Polymorphism (SNP)-based phylogenetic analysis was performed for comparative genomics. SNPs were detected *via* read-mapping against a reference genome using the ASA^3^P pipeline (Schwengers et al., 2019). MAUVE v.2.3.1 was used for whole genome alignments (Darling et al., 2010).

The carbapenemase-encoding plasmid incompatibility (Inc) type in genomes was identified when a contig contained the carbapenemase gene and the plasmid-Inc type sequences. These contigs and the plasmids closed by long-read sequencing were used as references for read mapping and blastN/P to determine plasmidic localization of carbapenemase gene in the other isolates.

The *C. freundii* type-strain ATCC 8090 (Accession number CP049015) was used as reference to identify sequence alterations of *ompC* and *ompF* genes encoding outer membrane porins of the *C. freundii* isolates.

## Results

### Bacterial Isolates Origin and Clinical Characterization

During the 3-year study-period, 56 multidrug-resistant *Citrobacter* spp. isolates were analyzed. Fifty-three (94.6%) of them produced carbapenemases and were obtained from 52 patients and 1 hospital environment sample of 14 German hospitals located across the State of Hesse. Except for one neonate, patients were adults and 77% were male. Most patients were elderly with a median of 70 years of age and 80% of them aged ≥ 60 years. Thirty-eight (73%) isolates were obtained from rectal swab samples. Fourteen (27%) were isolated from clinical specimens and induced clinical infections as follows: seven (50%) urinary tract infections, 2 (14%) wound infections, 1 (7%) bacteremia and 4 (29%) other infections (**Table 1**, **Supplementary Table S1**).

**Table 1:** Demographics of patients carrying carbapenemase-producing *Citrobacter* spp. Isolates.

Characteristics	Value for patient cases*
Mean age, yr. (range)	67 (<1 - 93)
Aged ≥ 60 yr.	41 (78.8%)
Male	40 (76.9%)
Female	12 (23.1%)
Sampling site	
Clinical specimen	14 (26.9%)
Rectal swab samples	38 (73.1%)
Clinical symptom	
Urinary tract infections	7 (50.0%)
Wound infections	2 (14%
Bacteraemia	1 (7%)
Other infections	4 (29%)

*n = 52. Data are no. (%) of patients unless otherwise indicated.

In four cases, the patients had poly-microbial cultures with other species in addition to *Citrobacter* spp. These being one case of KPC-2 *C. freundii* with an *E. coli;* one case of KPC-2 *C. koseri* with a *K. pneumoniae;* one case of OXA-48 *C. braakii* with an *E. coli* and a *K. pneumoniae;* and one case of VIM-1-positive *C. freundii* with a *K. michiganensis.* All these additional isolates carried the identical carbapenemase-type as the *Citrobacter* spp. isolates respectively (**Supplementary Table S1**).

### Genomic Characterization (Phylogeny, MLST, Carbapenemase, ARGs and Porins Genes)

The genome sequencing statistics of 56 carbapenem-resistant (CR) *Citrobacter* spp. isolates is summarized in the **Supplementary Table S2**. 53 (94.6%) isolates were carbapenemase-producing (CP) *Citrobacter* and three (5.4%) isolates did not produce any carbapenemase (designated as “non-carbapenemase-producers”, NCP).

**Figure 1:**
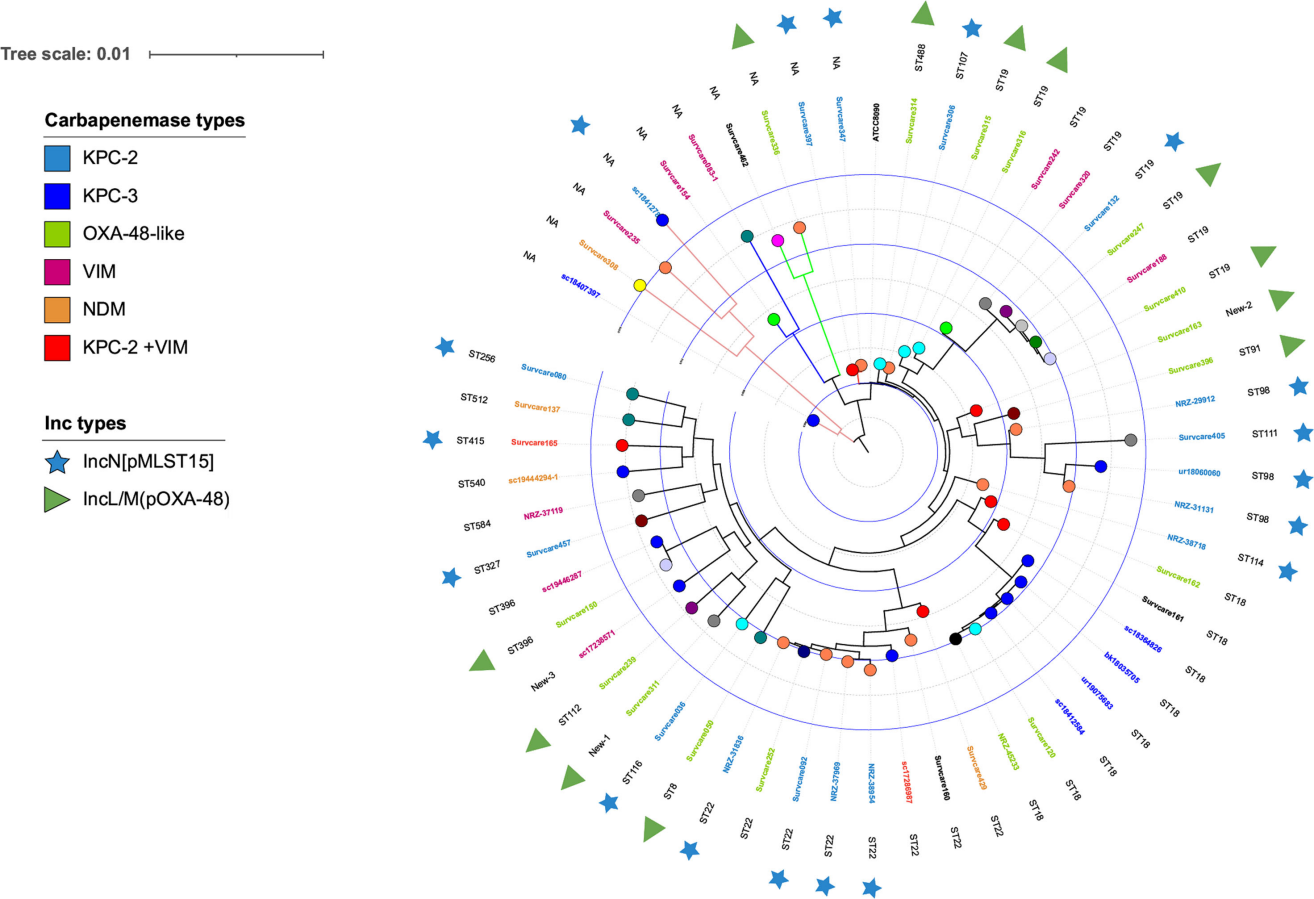
Phylogenetic tree of the 56 sequenced *Citrobacter* spp. isolates based on SNPs-analysis with the *C. freundii* strain ATCC 8090 (Access No. BBMV01000001) as reference genome, generated and annotated with the iTOL tool (https://itol.embl.de/tree/) (Letunic and Bork, 2021). The bacterial species is displayed with different branch line colors: *C. freundii*, black, *C. portucalensis*, orange, *C. europaeus*, blue, *C. braakii*, green, and *C. koseri*, red. The hospitals, from which the isolates were obtained, are indicated with the 16 different colored circles on the tree nodes. The different identified carbapenemase types are marked with different colors according to the isolate labels. ST, *C. freundii* MLST types of the *C. freundii* isolates. IncN[pMLST15]: the *bla*_KPC-2_ gene-carrying plasmid are represented by an asterisk in the KPC-2 carbapenemase-producing isolates. IncL/M(*pOXA-48*): the Inc type of plasmids identified in the OXA-48-like carbapenemase-positive isolates and likely to carry their *bla*_OXA-48_ or *bla*_OXA-162_.

Among the 53 CP *Citrobacter* spp. isolates 44 (83.0%) were *C. freundii*, four (7.5%) were *C. portucalensis*, two (3.8%) were *C. koseri*, two (3.8%) were *C. europaeus* and one (1.9%) was *C. braakii*. 51 (96%) isolates harbored a single carbapenemase, while the remaining two isolates (4%) possessed two different carbapenemases (**Figure 1**, **Supplementary Table S3**).

#### Citrobacter freundii

The phylogenetic analysis of the 44 C*. freundii* isolates revealed a broad genetic variability (**Figure 1**). Chromosomal Multi-Locus-Sequence Typing (MLST) identified 22 different genome STs. The most frequent ST types were ST19 and ST22, which were represented with 8 isolates each, followed by ST18 with 7 isolates and ST98 with 3 isolates. Interestingly, ST19 and ST22 isolates display an increase in detection frequency in the last two years of the study. This might point towards those two sequence types are fast emerging and might become predominant clones in the healthcare setting. The remaining 18 types represent single isolates.

In terms of antibiotic resistance genes (ARGs) content, the *C. freundii* isolates harbored ARGs against up to nine antibiotics classes, with up to 22 ARGs in a single isolate (**Supplementary Table S3**). The fraction of identified carbapenemase types KPC, OXA-48 group, VIM and NDM in the *C. freundii* isolates was 43%, 33%, 17% and 7%, respectively. The individual alleles were KPC-2 (n=16), OXA-48 (n=13), KPC-3 (n=4), VIM-1 (n=3), VIM-2 (n=3), VIM-4 (n=2), NDM-5 (n=2), OXA-162 (n=2) and NDM-1 (n=1) (**Figure 1**). Two *C. freundii* isolates harbored two different carbapenemases each (KPC-2 and VIM-1 or VIM-4) and were resistant to all three carbapenems tested.

In addition to the carbapenemase genes, all of the CP *C. freundii* isolates harbored other β-lactam-resistance genes. All *C. freundii* isolates carried chromosomal *bla*_CMY_, 29.5% carried *bla*_CTX-M_ genes including *bla*_CTX-M-15_, *bla*_CTX-M-9_, *bla*_CTX-M-3_ and *bla*_CTX-M-1_, 61.3% carried a *bla*_OXA-1_, *bla*_OXA-10_ or *bla*_OXA-17_, and 59.1% carried a *bla*_TEM-1B_ allele. Resistance genes to the antibiotic classes aminoglycosides, fluoroquinolones, sulphonamides, phenicols, trimethoprim, MLS (macrolide, lincosamide and streptogramin B), rifampicin, and tetracycline were detected in 93.2%, 86.4%, 86.4%, 81.8%, 75.0%, 61.4%, 56.8%, and 31.8% of the *C. freundii* isolates, respectively. Acquired resistance genes towards fosfomycin and colistin could not be detected in any isolates.

The amount of ARGs identified in the *C. freundii* genome other than the carbapenemase gene was not related to the ST type of the genome, but seems to be linked to the different types of carbapenemases carried by the isolates. It was highest in the KPC positive isolates with an average value of 15.2, followed by isolates carrying NDM (14.3), VIM (11.6) and OXA-48 (8.4).

No general relationship was found between the carbapenemase types and the genome types of the *C. freundii* isolates analyzed, with exception of KPC-3, which in the present study derived from a clonal ST18 *C. freundii* outbreak (**Figure 1**). The majority (almost 70%) of the carbapenemase-producing *C. freundii* differed in their ST types and occurred only once during the entire study period. In few cases, a ST type carrying an identical carbapenemase was repeatedly identified, namely a four-time occurrence of ST19-OXA-48, a three-time occurrence of ST18-OXA-48 and of ST98-KPC-2, each at different hospitals, while ST22-KPC-2 occurred four-times at a single hospital.

Each CP *C. freundii* isolate showed nucleotide alternations of the genes *ompC and ompF*, which encode the outer membrane porins, and harbored either an alternating *ompC* or *ompF*. An intact *ompC* identical to the type-strain ATCC 8090 was found in the genomes of three isolates and modified variants of *ompC* in the remaining 41 CP isolates. An intact *ompF* gene was identified in 24 isolates and an altered version, either by disruption or by replacement of several amino acid residues of *ompF*, was found in the other 30 isolates. Of the 44 CP *C. freundii* isolates, 17 showed modifications in both *ompC and ompF*, and the other 27 displayed an alternation in one of the two genes (**Supplementary Table S3**).

Two *C. freundii* isolates were NCP. These harbored a *bla*_CMY_ allele and a *bla*_TEM-1B_, plus either a *bla*_CTX-M-9_ or a *bla*_DHA-1_ (**Supplementary Table S3**). Furthermore, they contained several ARGs belonged to 7 different antibiotic classes (**Supplementary Table S3**). For the outer membrane porine-encoding genes, one NCP isolate showed alternations in *ompC* and a disrupted *ompF*, and the other one showed a changed *ompC* and a wild-type *ompF*.

#### Citrobacter braakii, C. europaeus, C. koseri and C. portucalensis

The phylogenic analysis revealed genetic dissimilarities between the isolates within the species *C. portucalensis, C. europaeus, C. braakii and C. koseri.* This, in combination with the different carbapenemases carried, suggests that almost all of them were sporadic occurrences (**Figure 1** and **Supplementary Table S3**).

Each *C. portucalensis* isolate (n=4) carried a different carbapenemase (KPC-2, KPC-3, NDM-5 or VIM-1) and a different chromosomal CMY-type *ampC* gene allele, and one of them carried additionally a *bla*_SHV-12_. However, the antimicrobial classes according to the ARGs contained were similar to the *C. freundii*. The two *C. europaeus* isolates were both VIM-1- producers and did not carry *bla*_CMY_, but *bla*_CFE-1_-type AmpC beta-lactamase, while the two *C. koseri* isolates were both KPC-2 carriers and did not harbor *bla*_CMY_, but a *bla*_CKO-1_ or *bla*_MAL-1_. The CP *C. braakii* isolate possessed a *bla*_OXA-48_ gene and a chromosomal *bla*_CMY-83_. The NCP *C. braakii* isolate harbored a *bla*_CMY-74_ and a *qnrB40*. (**Supplementary Table S3**).

### Antimicrobial Phenotypes

Antimicrobial susceptibility testing (AST) of the *Citrobacter* spp. isolates demonstrated that the 53 carbapenemase-producing isolates presented multi-drug-resistance (**Table 2**). All of the tested isolates were resistant to ampicillin, ampicillin/sulbactam, piperacillin, piperacillin/tazobactam, cefepim, cefuroxim and aztreonam. Resistance to ertapenem, imipenem and meropenem was 85%, 77% and 72%, respectively, of the isolates tested. However, none of them were phenotypically susceptible to all three carbapenems tested. For tigecycline 88% of the tested isolates were susceptible and 12% resistant.

**Table 2:** Results of the Antimicrobial susceptibility testing of 53 Carbapenemase-producing *Citrobacter* spp. isolates as determined by VITEK-2 and according to EUCAST breakpoints.

Antibiotic	No. of isolates resistant validated/tested	Resistant (%)	No. of isolates sensitive validated/tested		Sensitive (%)	
Ampicillin	53/53	100	0/53		0	
Ampicillin +Sulbactam	53/53	100	0/53		0	
Piperacillin	31/31	100	0/31		0	
Piperacillin +Tazobactam	50/50	100	0/50		0	
Cefepim	31/31	100	0/31		0	
Cefuroxim	14/14	100	0/14		0	
Cefpodoxim	52/53	98	1/53		2	
Cefotaxim	51/53	96	2/53		4	
Ceftazidim	51/53	96	2/53		4	
Ertapenem	35/41	85	6/41		15	
Imipenem	41/53	77	12/53		21	
Meropenem	38/53	72	15/53		29	
Aztreonam	33/33	100	0/33		0	
Ciprofloxaxin	42/53	79	11/53		21	
Moxifloxacin	37/42	88	5/42		12	
Ofloxacin	30/31	97	1/31		3	
Gentamicin	35/53	66	18/53		24	
Tigecycline	6/50	12	44/50		88	
Trimethoprim +Sulfamethoxazol	37/53	70	16/53		30	

Phenotypic differences of resistance to carbapenems, ciprofloxacin and gentamicin seem to be associated with the different carbapenemase types. The KPC-carbapenemase-producing *Citrobacter* isolates showed similar resistance rates to all three carbapenems tested, ertapenem, imipenem and meropenem, at approximately 86%. However, OXA-48 carbapenemase-producing isolates (included OXA-162) differed in resistance to these three agents, with 100% resistance to ertapenem (14/14), 40% resistance to imipenem (6/15) and 33% resistance to meropenem, respectively. Within the NDM or VIM carbapenemase- producers, 37.5% were resistant to ertapenem and 95% resistant to imipenem or meropenem. The resistance to ciprofloxacin was 86%, 75% and 67% observed for KPC-, MBL (NDM or VIM)-, and OXA-48 producers, respectively. To gentamicin, most of the KPC- (90%) and MBL-producers (92%) were resistant and most of the OXA-48 producers (~80%) were susceptible.

### Identification of Carbapenemase-Encoding Plasmids

To identify the genetic locations of the acquired ARGs, especially for the carbapenemase genes, we determined the plasmid content of each genome. For the *Citrobacter* spp. isolates sequenced plasmid incompatibility groups of IncN (n=22), ColRNAI (n=18), IncFII (n=16), IncFI (n=15), IncR (n=13), IncL/M (n=13), IncHI2 (n=9), IncHI1 (n=8), IncA/C2 (n=6), IncQ (n=6), TrfA (n=8), IncX (n=3), and one each of Col(BS512), IncN3, pKPC-CAV1321 and Rep were identified. All but one isolate genomes contained at least one of the identified plasmid types (**Supplementary Table S3**).

Among the IncN group, IncN[pMLST15] was most frequently identified (n=18). The remaining were each one of IncN[pMLST-7], IncN[pMLST-Unknown], IncN2A and IncN3.

**Figure 2:**
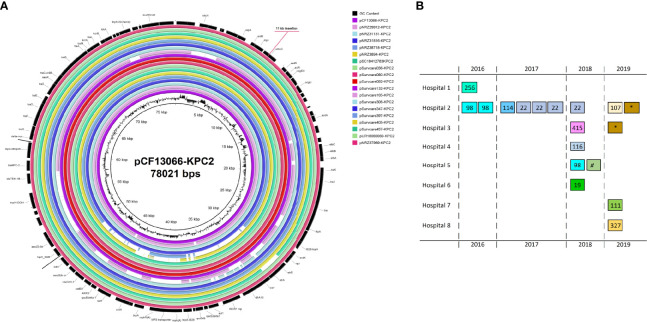
Genetic map of the completed genomes of the *bla*_KPC-2_-encoding IncN[pMLST15] **(A)** and the distribution of the IncN pMLST15 plasmids according to the time from 2016 to 2019 and hospitals as well as the genome types and species **(B)**. The plasmid pCF37969-KPC2 was almost identical to the early identified pCF13066-KPC2, which has been isolated from a multi-species carbapenem-resistant outbreak in 2014 in Hesse (separate study), and differed by a ~11 kb insertion. Each box in **(B)** indicates one isolate. The numbers in the boxes indicate the MLST-types of the *C. freundii* isolates as well as the symbol (*) for the species *C*. *koseri* and the symbol (^#^) for *C. portucalensis*.

The IncN[pMLST15] was found exclusively in the KPC-2-producing isolates and served as the most important genetic carrier for the KPC-2 gene (95% of KPC-2 isolates) (**Figure 1**). Remarkably, these plasmids identified in different species *C. freundii, C. koseri* and *C. portucalensis* and different genome ST types were almost identical. On these plasmids up to 13 different ARGs are located conferring resistance to 8 antibiotic classes: carbapenem and β-lactam (*bla*_KPC-2_, *bla*_TEM-1B_ and *bla*_OXA-1_); aminoglycoside (*aac(3)-IId*, *strA* and *strB*); fluoroquinolone (*aac(6’)Ib-cr*, *qnrB2 or qnrB1*); macrolide *mph(A*); phenicol *catB3*; rifampicin *ARR-3*; sulphonamide *sul1* and trimethoprim *dfrA18* (**Table 3**). In nine isolates, more than 90% of their total acquired ARGs were carried by the IncN[pMLST15]. The genetic structure surrounding *bla*_KPC-2_ on the IncN[pMLST15] plasmids was unique as described previously (Yao et al., 2014; Schweizer et al., 2019), and identical in 16 of 18 isolates. The genome of NRZ-37969 was completed by long-read re-sequencing which resulted in 6 contigs, one of them was the circular IncN[pMLST15] plasmid with a size of 89053 bps. The sequence was similar to the plasmid pCF13066-KPC2 (78021 bps), from an earlier study, but differed in size by an 11 kb insertion in the position 8100 according to the pCF13066-KPC-2 (**Figure 2**). Read mapping demonstrated all IncN[pMLST15] plasmids to be identical, concerning the backbone and the unique segments to those of other species e.g. *E. coli*, *K. pneumoniae*, *Enterobacter* spp. etc. (Data not shown).

**Table 3:** Distribution and genetic characteristics of the identified KPC-2-carrying IncN[pMLST15] plasmids among the *Citrobacter* spp. Isolates.

Isolate	Year	Hospital	Species	MLST-type of the isolate	Lengths, Copy-Number of the predicted IncN [pMLST-15] plasmid sequences and the encoded ARGs															Other Inc types	ARGs on Chromosome and other plasmids		
					Length (bps)*	Copy No. **	Beta-lactam			Aminoglycoside			Fluoroquinolone		other classes^#^								
Survcare080	2016	1	*C. freundii*	256	78021	1.84	*bla*_KPC-2_	*bla*_TEM-1B_	*bla*_OXA-1_	*aac(3)-IId*	*strA*	*strB*	*aac(6’)Ib-cr*	*qnrB2*	*mph(A)*	*catB3*	*ARR-3*	*sul1*	*dfrA18*			*bla*_CMY-51_ *, tet(A)*
NRZ-29912	2016	2	*C. freundii*	98	75302	0.98	*bla*_KPC-2_	*bla*_TEM-1B_	*bla*_OXA-1_	*aac(3)-IId*	*strA*	*strB*	*aac(6’)Ib-cr*	*qnrB2*	*mph(A)*	*catB3*	*ARR-3*	*sul1*	*dfrA18*	ColRNAI		*bla*_CMY-84_ *, qnrB38*
NRZ-31131	2016	2	*C. freundii*	98	77725	3.73	*bla*_KPC-2_	*bla*_TEM-1B_	*bla*_OXA-1_	*aac(3)-IId*	*strA*	*strB*	*aac(6’)Ib-cr*	*qnrB2*	*mph(A)*	*catB3*	*ARR-3*	*sul1*	*dfrA18*	ColRNAI		*bla*_CMY-84_ *, aadA1, qnrB38*
NRZ-38718	2017	2	*C. freundii*	114	61001	5.01	*bla*_KPC-2_	*bla*_TEM-1B_		*aac(3)-IId*			*aac(6’)Ib-cr*		*mph(A)*							*bla*_CMY-48_
NRZ-37969	2017	2	*C. freundii*	22	89053	0.69	*bla*_KPC-2_	*bla*_TEM-1B_	*bla*_OXA-1_	*aac(3)-IId*	*strA*	*strB*	*aac(6’)Ib-cr*	*qnrB2*	*mph(A)*	*catB3*	*ARR-3*	*sul1*	*dfrA18*	ColRNAI		*bla*_CMY-48_ *, aadA1, tet(A), dfrA1*
NRZ-31836	2017	2	*C. freundii*	22	78021	2.73	*bla*_KPC-2_	*bla*_TEM-1B_	*bla*_OXA-1_	*aac(3)-IId*	*strA*	*strB*	*aac(6’)Ib-cr*	*qnrB2*	*mph(A)*	*catB3*	*ARR-3*	*sul1*	*dfrA18*			*bla*_CMY-48_ *, aadA1*
NRZ-38954	2017	2	*C. freundii*	22	69402	0.59	*bla*_KPC-2_	*bla*_TEM-1B_		*aac(3)-IId*	*strA*	*strB*	*aac(6’)Ib-cr*		*mph(A)*	*catB3*	*ARR-3*	*sul1*	*dfrA18*	IncHI2[ST-1], IncHI2A		*bla*_CMY-48_ *, bla*_CTX-M-9_ *, aadB, dfrA1*
Survcare092	2018	2	*C. freundii*	22	78021	3.60	*bla*_KPC-2_	*bla*_TEM-1B_	*bla*_OXA-1_	*aac(3)-IId*	*strA*	*strB*	*aac(6’)Ib-cr*	*qnrB2*	*mph(A)*	*catB3*	*ARR-3*	*sul1*	*dfrA18*	IncHI2A		*bla*_CMY-48_ *, bla*_CTX-M-9_ *, aadA1, aadA2, aadB, tet(A), dfrA1*
Survcare306	2019	2	*C. freundii*	107	73409	4.05	*bla*_KPC-2_		*bla*_OXA-1_				*aac(6’)Ib-cr*	*qnrB1*	*mph(A)*	*catB3*	*ARR-3*	*sul1*		Col(BS512)		*bla*_CMY-48_ *, bla*_CTX-M-15_ *, aadB, aac(3)-IIa, catA1*
Survcare347	2019	2	*C. koseri*	NA	78021	1.73	*bla*_KPC-2_	*bla*_TEM-1B_	*bla*_OXA-1_	*aac(3)-IId*	*strA*	*strB*	*aac(6’)Ib-cr*	*qnrB2*	*mph(A)*	*catB3*	*ARR-3*	*sul1*	*dfrA18*	ColRNAI, IncFII, IncI1[ST-36]		*bla*_CKO-1_
Survcare397	2019	3	*C. koseri*	NA	78021	1.80	*bla*_KPC-2_	*bla*_TEM-1B_	*bla*_OXA-1_	*aac(3)-IId*	*strA*	*strB*	*aac(6’)Ib-cr*	*qnrB2*	*mph(A)*	*catB3*	*ARR-3*	*sul1*	*dfrA18*	ColRNAI		*bla*_MAL_1_
Survcare165	2018	3	*C. freundii*	415	~55098	2.60	*bla*_KPC-2_						*aac(6’)Ib-cr*	*qnrB6*			*ARR-3*	*sul1*		ColRNAI, IncFIB(K)		*bla*_CMY-48_ *, bla*_VIM-1_ *, aacA4, aadA1, aadA16, aph(3’)-Ia, aph(4)-Ia, aac(3)-IVa, dfrA27*
Survcare036	2018	4	*C. freundii*	116	78201	3.77	*bla*_KPC-2_	*bla*_TEM-1B_	*bla*_OXA-1_	*aac(3)-IId*	*strA*	*strB*	*aac(6’)Ib-cr*	*qnrB2*	*mph(A)*	*catB3*	*ARR-3*	*sul1*	*dfrA18*	IncHI2[ST-1]		*bla*_CMY-65_ *, bla*_DHA-1_ *, aac(6´)-IIc, ere(A), qnrB4*
ur18060060	2018	5	*C. freundii*	98	78021	4.30	*bla*_KPC-2_	*bla*_TEM-1B_	*bla*_OXA-1_	*aac(3)-IId*	*strA*	*strB*	*aac(6’)Ib-cr*	*qnrB2*	*mph(A)*	*catB3*	*ARR-3*	*sul1*	*dfrA18*	IncFIA(HI1)		*bla*_CMY-84_ *, qnrB38*
sc18412783	2019	5	*C. portucalensis*	NA	78021	1.11	*bla*_KPC-2_	*bla*_TEM-1B_	*bla*_OXA-1_	*aac(3)-IId*	*strA*	*strB*	*aac(6’)Ib-cr*	*qnrB2*	*mph(A)*	*catB3*	*ARR-3*	*sul1*	*dfrA18*	ColRNAI		*bla*_CMY-77_ *, qnrB42*
Survcare132	2018	6	*C. freundii*	19	78021	2.40	*bla*_KPC-2_	*bla*_TEM-1B_	*bla*_OXA-1_	*aac(3)-IId*	*strA*	*strB*	*aac(6’)Ib-cr*	*qnrB2*	*mph(A)*	*catB3*	*ARR-3*	*sul1*	*dfrA18*	IncFII-FI, TrfA		*bla*_CMY-41_ *, aadA2, aph(3´)-Ia, dfrA12*
Survcare405	2019	7	*C. freundii*	111	78201	3.13	*bla*_KPC-2_	*bla*_TEM-1B_	*bla*_OXA-1_	*aac(3)-IId*	*strA*	*strB*	*aac(6’)Ib-cr*	*qnrB2*	*mph(A)*	*catB3*	*ARR-3*	*sul1*	*dfrA18*	IncHI1B(CIT)		*bla*_CMY-65,_ *qnrB38*
Survcare457	2019	8	*C. freundii*	327	76016	2.40	*bla*_KPC-2_	*bla*_TEM-1B_	*bla*_OXA-1_	*aac(3)-IId*			*aac(6’)Ib-cr*	*qnrB2*	*mph(A)*	*catB3*	*ARR-3*	*sul1*	*dfrA18*	ColRNAI		*bla*_CMY-48_ *, bla*_CTX-M-15_ *, aac(6’)-If, aac(3)-IIa, tet(A)*

*The lengths of the predicted plasmid sequences here were derived from the better result of read-mappings to pNRZ-37969KPC2 (89053) from this study and the pCF13066KPC2 (78021 bps) from the previous study, that was assembled using

PacBio-Sequencing. The lengths with 78021 bps are closed sequences, corresponding to pCF13066KPC2. The other lengths are approximations.

**The copy number of the plasmid was calculated by dividing the coverage of the plasmid-representing genes (repA, traI, traJ and traK) by the coverage of the 7 Citrobacter freundii MLST genes or the coverage of the De novel Assembly of the species C. braakii and C. portucalensis

^#^represent five antibiotic classes, macrolide (mph(A)), phenicols (catB3), rifampicin (ARR-3), sulphonamide (sul1), and trimethoprim (dfrA18).

**Figure 3:**
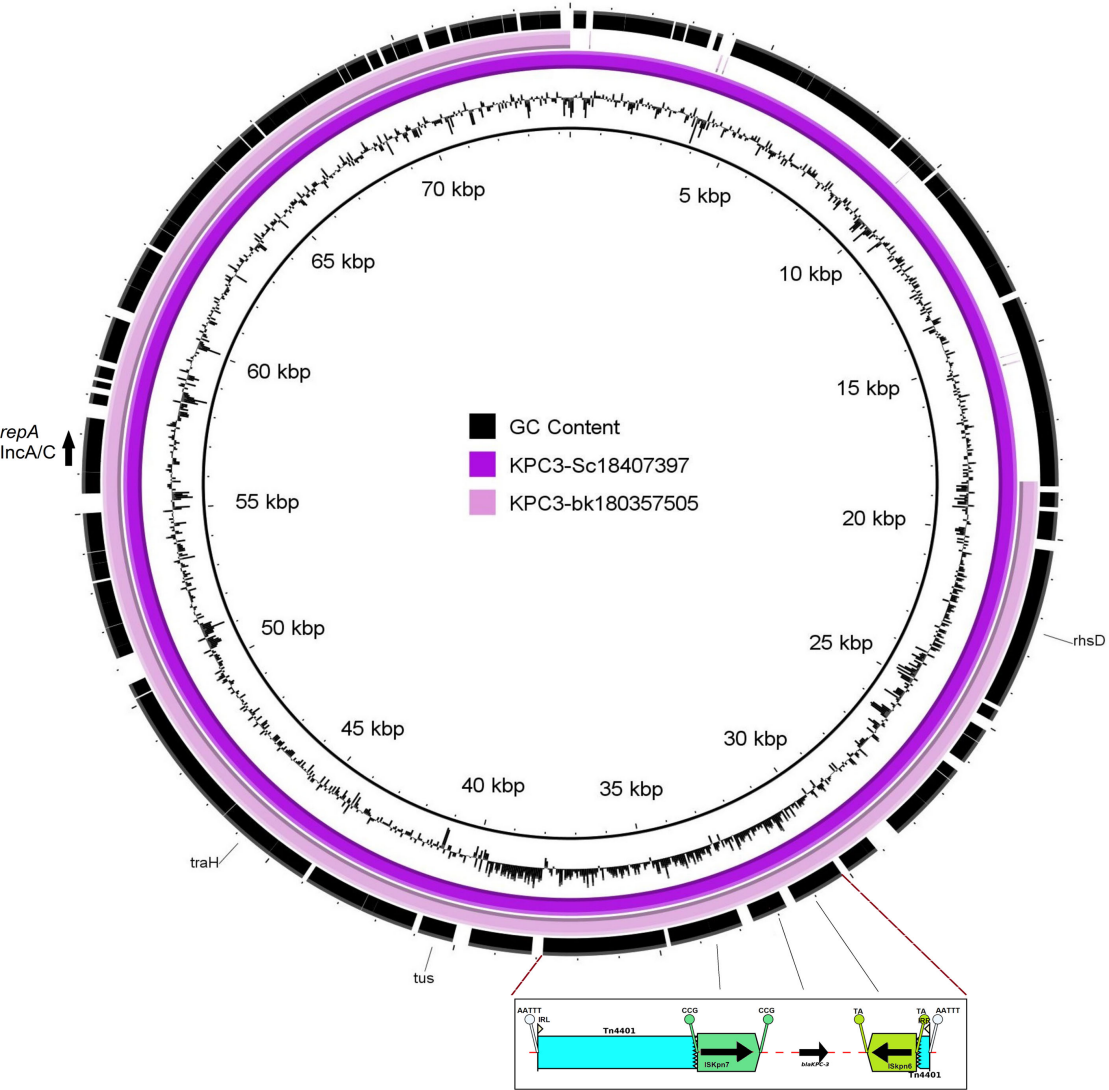
Genetic Map of IncA/C2 plasmid encoding a Tn*4401* associated *bla*_KPC-3_- carbapenemase.

In the five KPC**-**3-carrying isolates of species *C. freundii* (n=4) and *C. portucalensis* (n=1), plasmids of Inc group A/C2 were predicted. The *bla*_KPC-3_ gene and the IncA/C2 plasmid replication protein- encoding gene were co-located on a 56083 bps contig of the *C. freundii* isolate bk18035705 and a 74360 bps contig of *C. portucalensis* isolate sc18407397. Whereas the *bla*_KPC-3_ gene was flanked upstream with an insertion element IS*Kpn7* and downstream with an IS*Kpn6*. This element was associated with a Tn*4401* transposon (**Figure 3**).

**Figure 4:**
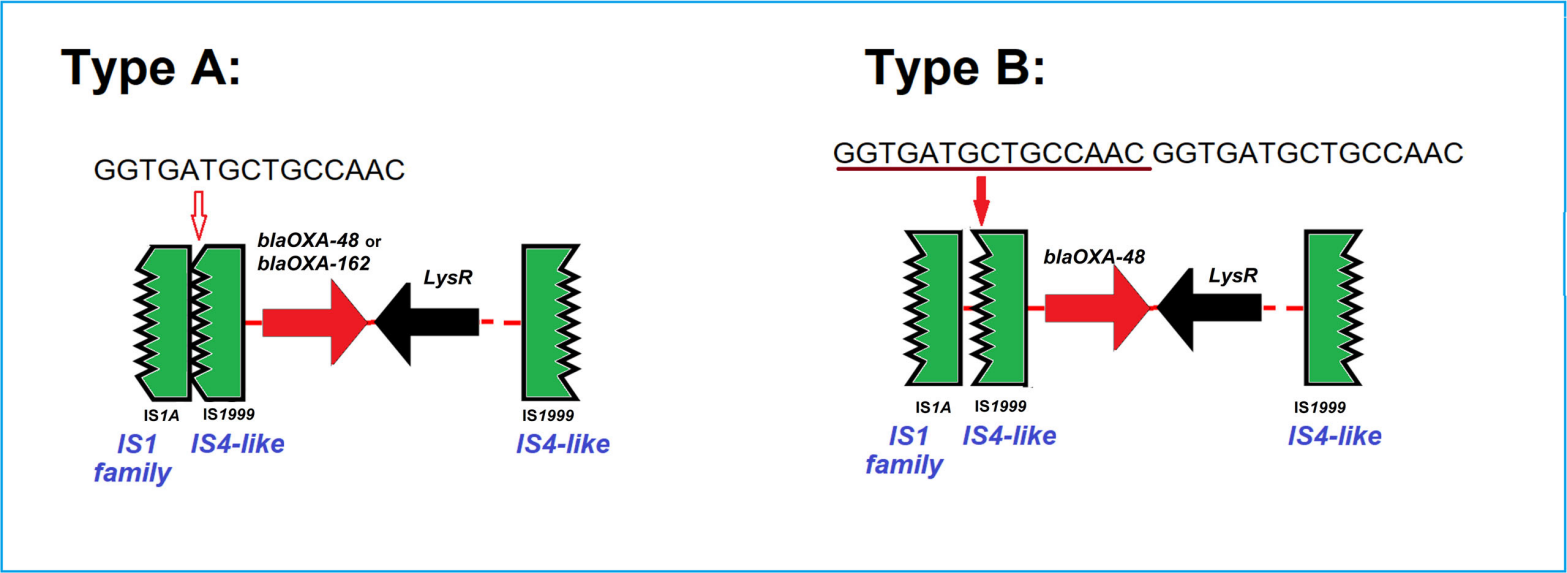
Two types of the genetic surroundings of the *bla*_OXA-48_ and *bla*_OXA-162_. Type A and B differ only in the presence or absence of a repetitive 15bp sequence (GGTGATGCTGCCAAC). Type A was detected in 9 *bla*_OXA-48_ or *bla*_OXA-162_-encoding IncL/M(*pOXA-48*) plasmids from 8 C*. freundii* isolates, i.e. Survcare050, Survcare150, Survcare247, Survcare311, Survcare315, Survcare316, Survcare396 and Survcare410 and one *C. braakii* Survcare336, as well as in the non-IncL/M(*pOXA-48*)-bearing Survcare252, which harbored a *bla*_OXA-48_ gene. Type B was present in four *bla*_OXA-48_-harboring isolates, whereas two isolates (Survcare163 and Survcare314) were associated with the IncL/M(*pOXA-48*) plasmid and two (Survcare162 and NRZ-45233) were not.

For ten of fourteen OXA-48 carbapenemase harboring isolates and both OXA-162 carbapenemase producing isolates, plasmids of the type IncL/M(*pOXA-48*) were identified. As we could not directly prove the *bla*_OXA-48_ plasmid location using short-read sequencing only, we analyzed the contigs (~2.5 kb) harboring *bla*_OXA-48_ to identify the genetic structures of the isolates and found a disrupted IS*1999* element typical for OXA-48. A 15 bps-insertion/repetition (GGTGATGCTGCCACC) between the disrupted IS elements, ΔIS*1A* and ΔIS*1999* downstream of *bla*_OXA-48_ was found for four isolates compared to the other eight isolates (**Figure 4**). The genetic structures surrounding the *bla*_OXA-162_ were identical to that of OXA-48 without the insertion. In addition, these isolates usually carried other plasmids, like IncFII, IncHI1, IncQ1 and IncR.

**Figure 5:**
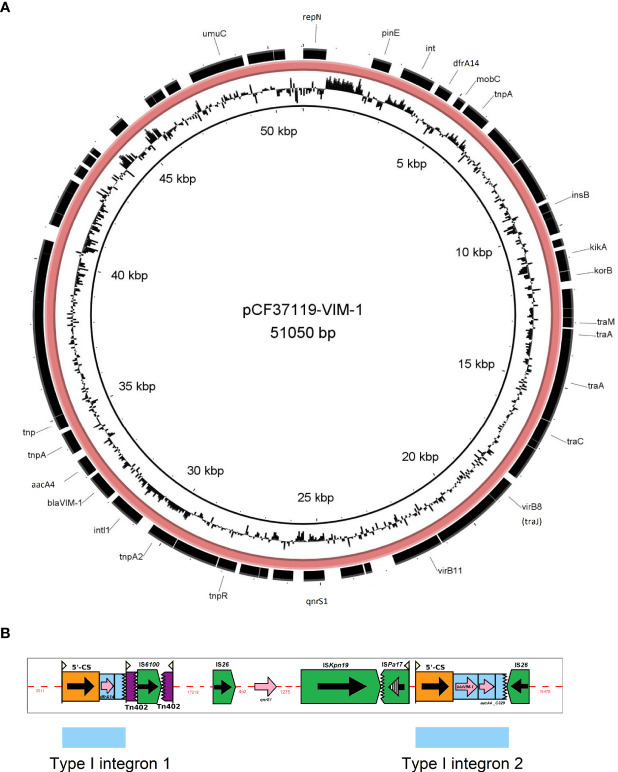
Genetic map of the complete genome of the *bla*_VIM-1_-encoding IncN[pMLST7] plasmid **(A)** and Depiction of insertion elements/transposons present in this plasmid **(B)**. The picture B was created using the Galileo AMR software (Partridge and Tsafnat, 2018).

We could demonstrate the presence of VIM-1-carrying IncN[pMLST7] plasmids in Survcare320 and NRZ-37119. The isolate NRZ-37119 underwent long-read-sequencing resulting in two circular complete sequences, a chromosome (5068647 bps) and a plasmid (51050 bps) of IncN[pMLST-7] with 3.78 coverage. The plasmid harbored four ARGs, *bla*_VIM-1_, *aacA4*, *qnrS1* and *dfrA14*. The *bla*_VIM-1_/*aacA4* (for resistance to aminoglycoside such like amikacin, gentamicin and tobramycin) and *dfrA14* genes were located in two different type I integrons disrupted either by an IS*26* element or by a Tn*402*/IS*6100* combination, respectively (**Figure 5**). In the chromosome sequence, *bla*_CMY-79_ and *tet(B)* were predicted (**Supplementary Table S3**).

## Discussion

In this study, we conducted a retrospective review of genomic typing and epidemiology of CR *Citrobacter* spp. from hospital settings in a medium sized State with 6.2 Mio habitants in Germany over a period of more than three years. We analyzed the genomes of 56 *Citrobacter* spp. in the context of a CRGN project consisting of 512 Enterobacterales isolates. We found that rates of carbapenemase-producing *Citrobacter* spp. (CPCs) did increase over the study period, with 10.1%, 16.7% and 14.3% in 2017, 2018 and 2019, respectively, and that 27% of patients, from whom the CPCs were isolated, suffered from a clinical infection in contrast to a colonization. Our data revealed a large genetic diversity of *Citrobacter* spp. in the study region suggesting that the emergence was not only due to clonal transfer. We also found that the carbapenem resistance phenotype was mostly mediated by the acquisition of carbapenemases and interestingly, the carbapenemase-producing fraction in the *Citrobacter* spp. (CPCs), was the highest among all CR Enterobacterales including species *Klebsiella pneumoniae*, *Escherichia coli* and *Enterobacte*r spp. in the same study period and region (Yao et al., unpublished data).

The current increase in CPCs, is in line with studies in Spain and the US, which indicates towards a sustained global trend (Arana et al., 2017a; Babiker et al., 2020). The patients´ carbapenem application was considered an associated risk factor in a long-term-study (Babiker et al., 2020). In our data, we found *Citrobacter freundii* as the predominant carbapenemase-producing species, which accounted for 83% of isolates, with the remaining 17% comprising of the species *C. portucalensis*, *C. koseri*, *C. europaeus*, *C. braakii*. The predominance of *C. freundii* and the distribution of other *Citrobacter* species were similar to previous studies (Arana et al., 2017b; Babiker et al., 2020). In our study, the *C. freundii* STs 19 and 22 seem to be emerging over the study period.

OXA-48 and VIM-1 are the most frequent carbapenemase types detected in Germany and *K. pneumoniae*, *E. coli* and *Enterobacter* spp. are the most affected species (Grundmann et al., 2017; Becker et al., 2018; Pfennigwerth, 2020). However, very little information is available about carbapenemase-producing *Citrobacter freundii* (Peter et al., 2014; Yao et al., 2014; Schweizer et al., 2019). Nine different carbapenemase types were detected among *Citrobacter* spp. isolates under study Their distribution, with a predominance of KPC-2 and OXA-48, differed substantially from studies in other countries or regions. While 59% VIM, 32% OXA-48 and 20% KPC-2 were found in the *Citrobacter* spp. in Spain, more than two-thirds KPC-3 were found in the isolates of *Citrobacter species* in a US study (Arana et al., 2017a; Babiker et al., 2020). This demonstrates that carbapenemase diversity varies according to the geographic location, as previously stated (Nordmann et al., 2011). We describe for the first time two *C. freundii* co-producing KPC-2 and either VIM-1 or VIM-4 carbapenemases in Germany. Some earlier studies have reported the co-production of two carbapenemases such as KPC-2 plus NDM-1 or VIM-1 plus OXA-48 in *C. freundii* and KPC-2 plus VIM-2 in *K. pneumoniae* (Feng et al., 2015; Falco et al., 2016; Arana et al., 2017a), but no *Citrobacter* spp. with KPC-2 and VIM. As acquisition of *mcr-1* resistance gene to the last-line antibiotic colistin was reported in *Citrobacter europaeus* from a healthy child, the presence of this gene represented a potential for expanding the ARGs profile (Giani et al., 2018). However, *mcr-1* could not be detected in the studied isolates.

In this study, 95% of the carbapenem-resistant *Citrobacter* spp. isolates were found to have carbapenemase-encoding genes, a rate higher than that reported in prior studies, which was roughly two-thirds (Arana et al., 2017a; Babiker et al., 2020). This was also higher than that of other Enterobacterales species e.g. *E. coli*, *Klebsiella* spp. and *Enterobacter* spp. in the same study period. This could be explained by the fact that in the studied isolates a high diversity of plasmids and a relatively high proportion of carbapenemase-encoding plasmids was found. In addition, plasmid-mediated carbapenemase transmission between *Citrobacter* spp. and other bacterial species could be demonstrated when the patients carried multiple bacteria with the same carbapenemase-encoding plasmids, such as the four cases mentioned above.

The analysed *Citrobacter* spp. isolates carried up to 22 ARGs, exclusive the carbapenemase genes reflecting their MDR phenotypes. This impressing accumulation of resistance traits is exceptional, compared to other Enterobacterales and might point towards these *Citrobacter* becoming a relevant reservoir of potentially transmissible resistance in the healthcare-setting. We predicted a few known-virulence genes such as an *astA-like* gene in one of the two OXA-162 positive *C. freundii* isolates as well as the cloacin-encoding gene *ccl* and the plasmid-encoded enterotoxin- encoding gene *senB* in the both *C. koseri* isolates.

All CR *Citrobacter* isolates, including the Non-carbapenemase-producing isolates harboured alternation/disruption of at least one gene of the outer membrane porine-encoding genes *ompC* and *ompF*. The absence of mutations or the presence of both intact genes *ompC* and *ompF* in all carbapenem-susceptible isolates (Arana et al., 2017a) conversely hints that these mutations could indirectly contribute to reduced susceptibility to carbapenems.

The analyses of the surrounding genetic structure of the carbapenemase-encoding genes and the identified respective Inc types of the carbapenemase-carrying plasmids in the *Citrobacter* spp. isolates suggests that about 70% of the identified carbapenemases could be characterized into three Inc groups, IncN, IncL/M and IncA/C2. We identified a KPC-3-encoding IncA/C2 plasmid for all five KPC-3 positives isolates, a KPC-2- encoding IncN[pMLST15] plasmid for 18 of 19 KPC-2-producing isolates and pMLST-7 IncN plasmids for two of six VIM-1-producing isolates. Furthermore, we predicted the IncL/M[*pOXA-48*] plasmid type for 12 of the 16 OXA-48 and OXA-162- carrying isolates.

The KPC-2-encoding IncN[pMLST15]-carrying isolates originated from eight different hospitals during a time period from 2016 to 2019 (**Figure 2B**). The whole-genome types of these isolates were heterogeneous. However, the mutually present KPC-2 IncN[pMLST15] plasmids were almost identical and occurred across the entire study period, independently of hospital-settings and bacterial genome types. The plasmid sequences were also highly homolog to a plasmid which had mediated a previous KPC-2 multi-species nosocomial outbreak in Hesse in 2014. In this context, we could observe that a patient who was involved in this previous outbreak was colonized with KPC-2 positive *C. freundii* harboring the same plasmid for a long period of time after the outbreak (first sample May 2014, followed Dec. 2014, June and Sep. 2015, Feb and June 2016) in the follow-up screening. It is particularly noteworthy that KPC-2 *Citrobacter* isolates with the same plasmid were consistently identified in this hospital during this study period. Furthermore, in a separate study, this plasmid was repetitively identified in different species (Yao et al., 2021 unpublished data). The *Citrobacter* spp. especially *C. freundii* seem to be frequent carriers of this plasmid and might even represent a specific reservoir. At least in our setting this plasmid seems to be both endemic and emerging and might be classified as a high-risk plasmid in terms of CR dissemination across different bacterial species. The reasons for this are difficult to determine and this impression might be biased by the study parameters. Therefore, further studies are needed to determine whether carbapenemase-producing *Citrobacter* spp. act as an intermediate reservoir to spread antibiotic resistance determinants to more virulent bacterial species or represent a more permanent reservoir for drug resistance accumulation with the risk of becoming pan-drug resistant pathogens themselves, and whether this multi-resistant KPC-2-bearing plasmid provides additional fitness advantage to the host bacteria.

## Conclusion

Our study provides a molecular-epidemiological overview on CR *Citrobacter* spp. originating from hospitals in Hesse, a midrange federal state of Germany according to population size and geographical area, over a three-year time period, based on whole-genome analyses. To the best of our knowledge, this study was the first of its kind in Germany. We detected a moderate increase in CR *Citrobacter* spp. numbers between 2016 and 2019 with carbapenemase production being the most prevalent mechanism for carbapenem resistance (95%). Our data revealed several additional findings: (i). clonal and plasmid-mediated polyclonal and interspecies spread of carbapenemase-producing *Citrobacter* spp. across the region in different hospitals; (ii). Carriage of nine different carbapenemase enzymes of the groups KPC, OXA-48, NDM and VIM by five species of *Citrobacter* spp., especially *C. freundii* that comprised a large genetic heterogeneity; however, emerging sequence types could be demonstrated (iii). Genetic locations of almost carbapenemases on plasmids and other genetic mobile elements evidencing that the studied *Citrobacter* spp. acquired carbapenem resistance *via* horizontal gene transfer; (iv). Detection of two potential high-risk *C. freundii* clones ST19 and ST22; and (v). Several specific plasmid types for carbapenemase-carriage, e.g. the IncN[pMLST15] for KPC-2, have been determined. In conclusion, the points towards *Citrobacter* spp. being an emerging bacterial species in terms of Carbapenemase carriage, resistance accumulation and potential transmission of resistance traits within the hospital setting. The *Citrobacter* spp. isolates, while usually not regarded as a typical human colonizer or nosocomial pathogen, display an above-average propensity for resistance accumulation of mobile and transferable resistance traits, thereby potentially representing a still underestimated reservoir for CR dissemination. Thus, the carbapenem-resistant *Citrobacter* spp. are an emerging health care and public health challenge which must be studied further to determine their role in resistance accumulation, dissemination and persistence in particular concerning carbapenem resistance in the hospital but also in the general population one health context. Further specific genomic surveillance studies in the mentioned settings seem to be mandatory.

## Data Availability Statement

The datasets presented in this study can be found in online repositories. The names of the repository/repositories and accession number(s) can be found below: https://www.ncbi.nlm.nih.gov/, BioProject PRJNA692829.

## Ethics Statement

Ethical approval was sought at the Ethics Committee of the State Medical Association of Hesse in Frankfurt/Main. The Committee decided on the 24th of January 2018, that an ethical approval for the project was not necessary, as for this study patient data were rendered anonymous.

## Author Contributions

YY, TC, and CI designed the study. AH, PH, YY, CI, TC, JF, LF, and ED collected the data and samples. AH, PH, JF, LF, CI, TC, and ED performed phenotyping and WGS of the isolates. YY, AH, PH, CI, TC, JF, LF, and ED analyzed the data. YY and JF performed the Genomic analyses. YY, CI, and TC wrote the manuscript that was critically reviewed and approved by all authors. All authors contributed to the article and approved the submitted version.

## Funding

This work was supported by the Bundesministerium fuer Bildung und Forschung (BMBF, Germany) within the German Center for Infection Research (DZIF/grant numbers, 8032808811, 8032808818, 8032808820 to TC/CI). Support was also obtained from the Hessian State Ministry for Social Affairs and Integration (HMSI) within the project SurvCARE Hessen and the Hessian Ministry of Higher Education, Research and Arts within the project HuKKH (Hessisches universitaeres Kompetenzzentrum Krankenhaushygiene). The funders of the study had no role in study design, data collection and analysis, decision to publish, or preparation of the manuscript.

## Conflict of Interest

The authors declare that the research was conducted in the absence of any commercial or financial relationships that could be construed as a potential conflict of interest.

## Publisher’s Note

All claims expressed in this article are solely those of the authors and do not necessarily represent those of their affiliated organizations, or those of the publisher, the editors and the reviewers. Any product that may be evaluated in this article, or claim that may be made by its manufacturer, is not guaranteed or endorsed by the publisher.

## References

[B1] Arana D. M. Ortega A. González-Barberá E. Lara N. Bautista V. Gómez-Ruíz D.. (2017a). Carbapenem-Resistant Citrobacter Spp. Isolated in Spain From 2013 to 2015 Produced a Variety of Carbapenemases Including VIM-1, OXA-48, KPC-2, NDM-1 and VIM-2. J. Antimicrob. Chemother. 72, 3283–3287. doi: 10.1093/jac/dkx325 29029114

[B2] Arana D. M. Ortega A. González-Barberá E. Lara N. Bautista V. Gómez-Ruíz D.. (2017b). Carbapenem-Resistant Citrobacter Spp. Isolated in Spain From 2013 to 2015 Produced a Variety of Carbapenemases Including VIM-1, OXA-48, KPC-2, NDM-1 and VIM-2. J. Antimicrob. Chemother. 72, 3283–3287. doi: 10.1093/jac/dkx325 29029114

[B3] Babiker A. Evans D. R. Griffith M. P. McElheny C. L. Hassan M. Clarke L. G.. (2020). Clinical and Genomic Epidemiology of Carbapenem- Nonsusceptible Citrobacter Spp. At a Tertiary Health Care Center Over 2 Decades. J. Clin. Microbiol. 58 (9), e00275–20. doi: 10.1128/JCM.00275-20 PMC7448640 32554477

[B4] Becker L. Kaase M. Pfeifer Y. Fuchs S. Reuss A. von Laer A.. (2018). Genome-Based Analysis of Carbapenemase-Producing Klebsiella Pneumoniae Isolates From German Hospital Patients 2008-2014. Antimicrob. Resist. Infect. Control. 7, 62. doi: 10.1186/s13756-018-0352-y PMC5930415 29744043

[B5] Brolund A. Lagerqvist N. Byfors S. Struelens M. J. Monnet D. L. Albiger B.. (2019). Worsening Epidemiological Situation of Carbapenemase-Producing Enterobacteriaceae in Europe, Assessment by National Experts From 37 Countries, July 2018. Eurosurveillance 24 (9), 1900123. doi: 10.2807/1560-7917.ES.2019.24.9.1900123 PMC6402177 30862330

[B6] Darling A. E. Mau B. Perna N. T. (2010). Progressivemauve: Multiple Genome Alignment With Gene Gain, Loss and Rearrangement. PloS One 5, e11147. doi: 10.1371/journal.pone.0011147 20593022 PMC2892488

[B7] Falco A. Ramos Y. Franco E. Guzmán A. Takiff H. (2016). A Cluster of KPC-2 and VIM-2-Producing Klebsiella Pneumoniae ST833 Isolates From the Pediatric Service of a Venezuelan Hospital. BMC Infect. Dis. 16 (1), 595. doi: 10.1186/s12879-016-1927-y PMC5075218 27770796

[B8] Falgenhauer L. Nordmann P. Imirzalioglu C. Yao Y. Falgenhauer J. Hauri A. M.. (2020). Cross-Border Emergence of Clonal Lineages of ST38 Escherichia Coli Producing the OXA-48-Like Carbapenemase OXA-244 in Germany and Switzerland. Int. J. Antimicrob. Agents 56 (6), 106157. doi: 10.1016/j.ijantimicag.2020.106157 32919009

[B9] Feng J. Qiu Y. Yin Z. Chen W. Yang H. Yang W.. (2015). Coexistence of a Novel KPC-2-Encoding MDR Plasmid and an NDM-1-Encoding Pndm-HN380-Like Plasmid in a Clinical Isolate of Citrobacter Freundii. J. Antimicrob. Chemother. 70, 2987–2991. doi: 10.1093/jac/dkv232 26260129

[B10] Giani T. Sennati S. Antonelli A. Di Pilato V. Di Maggio T. Mantella A.. (2018). High Prevalence of Carriage of Mcr-1-Positive Enteric Bacteria Among Healthy Children From Rural Communities in the Chaco Region, Bolivia, September to October 2016. Eurosurveillance 23 (45), 1800115. doi: 10.2807/1560-7917.ES.2018.23.45.1800115 PMC6234532 30424831

[B11] Grundmann H. Glasner C. Albiger B. Aanensen D. M. Tomlinson C. T. Andrasević A. T.. (2017). Occurrence of Carbapenemase-Producing Klebsiella Pneumoniae and Escherichia Coli in the European Survey of Carbapenemase-Producing Enterobacteriaceae (Euscape): A Prospective, Multinational Study. Lancet Infect. Dis. 17, 153–163. doi: 10.1016/S1473-3099(16)30257-2 27866944

[B12] Hu Y. Wang Y. Sun Q. Huang Z.-X. Wang H.-Y. Zhang R.. (2017). Colistin Resistance Gene Mcr-1 in Gut Flora of Children. Int. J. Antimicrob. Agents 50, 593–597. doi: 10.1016/j.ijantimicag.2017.06.011 28668691

[B14] Letunic I. Bork P. (2021). Interactive Tree of Life (Itol) V5: An Online Tool for Phylogenetic Tree Display and Annotation. Nucleic Acids Res. 49 (w6), w293–w296. doi: 10.1093/nar/gkab301 33885785 PMC8265157

[B15] Lipsky B. A. Hook Iii E. W. Smith A. A. Plorde J. J. (1980) Citrobacter Infections in Humans: Experience at the Seattle Veterans Administration Medical Center and a Review of the Literature. doi: 10.1093/clinids/2.5.746 6763304

[B17] Liu L. Lan R. Liu L. Wang Y. Zhang Y. Wang Y.. (2017). Antimicrobial Resistance and Cytotoxicity of Citrobacter Spp. In Maanshan Anhui Province, China. Front. Microbiol. 8, 1357. doi: 10.3389/fmicb.2017.01357 28775715 PMC5518651

[B16] Liu L. H. Wang N. Y. Wu A. Y. J. Lin C. C. Lee C. M. Liu C. P. (2018). Citrobacter Freundii Bacteremia: Risk Factors of Mortality and Prevalence of Resistance Genes. J. Microbiol. Immunol. Infect. 51, 565–572. doi: 10.1016/j.jmii.2016.08.016 28711438

[B18] Meier-Kolthoff J. P. Göker M. (2019). TYGS is an Automated High-Throughput Platform for State-of-the-Art Genome-Based Taxonomy. Nat. Commun. 10 (1), 2182. doi: 10.1038/s41467-019-10210-3 PMC6522516 31097708

[B19] Mohanty S. Singhal R. Sood S. Dhawan B. Kapil A. Das B. K. (2007). Citrobacter Infections in a Tertiary Care Hospital in Northern India. J. Infect. 54, 58–64. doi: 10.1016/j.jinf.2006.01.015 16815552

[B20] Nordmann P. Naas T. Poirel L. (2011). Global Spread of Carbapenemase Producing Enterobacteriaceae. Emerg. Infect. Dis. 17, 1791–1798. doi: 10.3201/eid1710.110655 22000347 PMC3310682

[B21] Oyeka M. Antony S. (2017). Citrobacter Braakii Bacteremia: Case Report and Review of the Literature. Infect. Disord. - Drug Targets 17, 59–63. doi: 10.2174/1871526516666161005155847 27658860

[B22] Partridge S. R. Tsafnat G. (2018). Automated Annotation of Mobile Antibiotic Resistance in Gram-Negative Bacteria: The Multiple Antibiotic Resistance Annotator (MARA) and Database. J. Antimicrob. Chemother. 73, 883–890. doi: 10.1093/jac/dkx513 29373760

[B23] Peter S. Wolz C. Kaase M. Marschal M. Schulte B. Vogel W.. (2014). Emergence of Citrobacter Freundii Carrying IMP-8 Metallo-β-Lactamase in Germany. New Microbes New Infect. 2, 42–45. doi: 10.1002/nmi2.36 25356340 PMC4184589

[B24] Pfennigwerth N. (2020). Bericht Des Nationalen Referenzzentrums Für Gramnegative Krankenhauserreger Zeitraum 1. Januar 2019 Bis 31. Dezember 2019. RKI. Epidemiol. Bull. 29, 3–10. doi: 10.25646/692

[B25] Pitout J. D. D. Peirano G. Kock M. M. Strydom K. A. Matsumura Y. (2020). The Global Ascendency of OXA-48-Type Carbapenemases. Clin. Microbiol. Rev. 33 (1), e00102–19. doi: 10.1128/CMR.00102-19 PMC6860007 31722889

[B13] Robert Koch-Institute (2016). Ifsg-Meldepflicht-Anpassungsverordnung: Zur Umsetzung Der Neuen Meldepflichten. Epidemiologisches Bulletin 2016 (4), 135–136. doi: 10.17886/EpiBull-2016-026

[B26] Schweizer C. Bischoff P. Bender J. Kola A. Gastmeier P. Hummel M.. (2019). Plasmid-Mediated Transmission of KPC-2 Carbapenemase in Enterobacteriaceae in Critically Ill Patients. Front. Microbiol. 10, 276. doi: 10.3389/fmicb.2019.00276 30837980 PMC6390000

[B27] Schwengers O. Hoek A. Fritzenwanker M. Falgenhauer L. Hain T. Chakraborty T.. (2020). ASA3P: An Automatic and Scalable Pipeline for the Assembly, Annotation and Higher Level Analysis of Closely Related Bacterial Isolates. PLos Comput. Biol. 16 (3), e1007134. doi: 10.1371/journal.pcbi.1007134 PMC7077848 32134915

[B28] Villa L. Carattoli A. Nordmann P. Carta C. Poirel L. (2013). Complete Sequence of the Inct-Type Plasmid Pt-OXA-181 Carrying the Blaoxa-181 Carbapenemase Gene From Citrobacter Freundii. Antimicrob. Agents Chemother. 57, 1965–1967. doi: 10.1128/AAC.01297-12 23357767 PMC3623325

[B29] Wang X. Chen G. Wu X. Wang L. Cai J. Chan E. W.. (2015). Increased Prevalence of Carbapenem Resistant Enterobacteriaceae in Hospital Setting Due to Cross-Species Transmission of the Bla NDM-1 Element and Clonal Spread of Progenitor Resistant Strains. Front. Microbiol. 6, 595. doi: 10.3389/fmicb.2015.00595 26136735 PMC4468908

[B30] Yao Y. Imirzalioglu C. Hain T. Kaase M. Gatermann S. Exner M.. (2014). Complete Nucleotide Sequence of a Citrobacter Freundii Plasmid Carrying KPC-2 in a Unique Genetic Environment. Genome Announc. 2 (6), e01157–14. doi: 10.1128/genomeA.01157-14 PMC4241661 25395635

[B31] Yao Y. Lazaro-Perona F. Falgenhauer L. Valverde A. Imirzalioglu C. Dominguez L.. (2017). Insights Into a Novel Blakpc-2-Encoding Incp-6 Plasmid Reveal Carbapenem-Resistance Circulation in Several Enterobacteriaceae Species From Wastewater and a Hospital Source in Spain. Front. Microbiol. 8, 1143. doi: 10.3389/fmicb.2017.01143 28702005 PMC5487458

