# Supplementary material for: Carbapenem-Resistant Citrobacter spp. as an Emerging Concern in the Hospital-Setting: Results From a Genome-Based Regional Surveillance Study
Source: Front Cell Infect Microbiol. 2021 Nov 11;11:744431. doi: 10.3389/fcimb.2021.744431 (PMC8632029; doi:10.3389/fcimb.2021.744431)
Supplement: Supplementary file 1 [file DataSheet_1.pdf]

Supplementary Table S1. Origin of the carbapenem-resistant *Citrobacter spp.* isolates

Isolate ID	Patient age	Gender	Year	Hospital ID	Species	Carbapenemase producer	Culture site	Clinical syndrome	Outbreak associated	Polymicrobial culture
bk18035705	79	F	2018	3770	*C. freundii*	*yes*	Blood	Infection	I	
NRZ_29912	72	F	2016	3331	*C. freundii*	*yes*	Rectal swab	Colonization		
NRZ-31131	75	M	2016	3331	*C. freundii*	*yes*	Rectal swab	Colonization		
NRZ-31836	72	M	2017	3331	*C. freundii*	*yes*	Rectal swab	Colonization		
NRZ-37119	64	M	2017	3001	*C. freundii*	*yes*	Swab screening	Colonization		*K. michiganensis*
NRZ-37969	73	M	2017	3331	*C. freundii*	*yes*	Rectal swab	Colonization		
NRZ-38718	82	F	2017	3331	*C. freundii*	*yes*	Rectal swab	Colonization		
NRZ-38954	59	M	2017	3331	*C. freundii*	*yes*	Rectal swab	Colonization		
NRZ-45233	89	F	2018	3000	*C. freundii*	*yes*	Urine	Infection	II	
sc17238571	52	M	2017	3770	*C. freundii*	*yes*	Rectal swab	Colonization		
sc17286987	46	M	2017	3770	*C. freundii*	*yes*	Rectal swab	Colonization	I	
sc18364826	63	M	2018	3770	*C. freundii*	*yes*	Rectal swab	Colonization	I	
sc18407397	59	F	2018	3770	*C. portucalensis*	*yes*	Rectal swab	Colonization		
sc18412584	68	M	2018	3770	*C. freundii*	*yes*	Rectal swab	Colonization		
sc18412783	68	M	2018	3770	*C. portucalensis*	*yes*	Rectal swab	Colonization		
sc19444294	80	M	2019	3770	*C. freundii*	*yes*	Nose throat swab	Colonization		
sc19446287	93	F	2019	3770	*C. freundii*	*yes*	Rectal swab	Colonization		
Survcare036	63	M	2018	3560	*C. freundii*	*yes*	Rectal swab	Colonization		
Survcare050	44	F	2017	3750	*C. freundii*	*yes*	Abscess swab	Infection		
Survcare080	69	M	2016	3750	*C. freundii*	*yes*	Rectal swab	Colonization		
Survcare083-1	74	M	2016	3750	*C. freundii*	*yes*	Groin swab	Colonization		
Survcare092	75	M	2018	3331	*C. freundii*	*yes*	Rectal swab	Colonization	I	
Survcare120	61	M	2018	3560	*C. freundii*	*yes*	Abdominal swab	Infection	II	
Survcare132	70	M	2018	3430	*C. freundii*	*yes*	Rectal swab	Colonization		
Survcare137	66	F	2018	3750	*C. freundii*	*yes*	Rectal swab	Colonization		
Survcare150	68	M	2018	3941	*C. freundii*	*yes*	Rectal swab	Colonization		
Survcare154	58	M	2018	3520	*C. freundii*	*yes*	Rectal swab	Colonization		
Survcare160	77	M	2018	3331	*C. freundii*	*not*	Rectal swab	Colonization		
Survcare161	75	M	2018	4000	*C. freundii*	*not*	Urine	Infection	II	
Survcare162	75	M	2018	4000	*C. freundii*	*yes*	Urethral swab	Infection	II	
Survcare163	61	M	2018	4000	*C. freundii*	*yes*	Intra-abdominale	Infection	II	
Survcare165	65	M	2018	4000	*C. freundii*	*yes*	Urine	Infection	II	
Survcare188	19	M	2018	3521	*C. freundii*	*yes*	Rectal swab	Colonization		
Survcare235	80	F	2019	3331	*C. portucalensis*	*yes*	Rectal swab	Colonization		
Survcare239	51	M	2019	3430	*C. freundii*	*yes*	Rectal swab	Colonization		
Survcare242	61	M	2019	3520	*C. freundii*	*yes*	transurethral catheter	Not variable data		
Survcare247	76	M	2019	3060	*C. freundii*	*yes*	Swab perineum	Not variable data		
Survcare252	85	M	2019	3820	*C. freundii*	*yes*	Rectal swab	Colonization	II	
Survcare306	78	M	2019	3331	*C. freundii*	*yes*	Rectal swab	Colonization		
Survcare308	0	M	2019	3330	*C. portucalensis*	*yes*	Rectal swab	Colonization		
Survcare311	79	F	2019	3001	*C. freundii*	*yes*	Rectal swab	Colonization		
Survcare314	90	M	2019	3560	*C. freundii*	*yes*	Rectal swab	Colonization	III	
Survcare315	66	M	2019	3560	*C. freundii*	*yes*	Rectal swab	Colonization	III	
Survcare316			2018	3560	*C. freundii*	*yes*	Hospital environment		III	
Survcare320	65	M	2019	3001	*C. freundii*	*yes*	Wound swab foot	Infection		
Survcare336	74	M	2019	3331	*C. braakii*	*yes*	Rectal swab	Colonization		*K. pneumoniae and E. coli*
Survcare347	58	M	2019	3331	*C. koseri*	*yes*	Rectal swab	Colonization		
Survcare396	82	F	2019	3300	*C. freundii*	*yes*	Rectal swab	Colonization		
Survcare397	79	M	2019	4000	*C. koseri*	*yes*	Rectal swab	Colonization		*K. pneumoniae*
Survcare405	71	M	2019	3001	*C. freundii*	*yes*	Tracheal secretions	Infection		*E. coli*
Survcare410	85	M	2019	3941	*C. freundii*	*yes*	Rectal swab	Colonization		
Survcare429	68	M	2019	4000	*C. freundii*	*yes*	Rectal swab	Colonization		
Survcare457	70	F	2019	3300	*C. freundii*	*yes*	Rectal swab	Colonization		
Survcare462	64	F	2019	3821	*C. braakii*	*not*	Rectal swab	Colonization		
ur18060060	56	M	2018	3770	*C. freundii*	*yes*	Urine	Infection	I	
ur19075683	78	M	2019	3770	*C. freundii*	*Yes*	Urine	Not variable data		

Supplementary Table S2. Genome sequencing statistics and accessions

Isolate	Seq-Platform	No. Reads	Average read length	No. Contigs	N50 (bp)	Genome Size (bp)	GC Content (%)	Coverage	BioSample	Accession
bk18035705	MiSeq	2030010	157.5	52	2550858	5092383	52	62.7	SAMN17371780	JAFHMA000000000
NRZ-29912	MiSeq	972598	119.6	379	25974	4964824	52	23.4	SAMN17371798	JAFHMS000000000
NRZ-31131	MiSeq	1463586	117,0	246	50209	4967061	52	34.4	SAMN17371802	JAFHMW000000000
NRZ-31836	NextSeq	4072040	132.7	103	189947	5184198	52	104.2	SAMN17371803	JAFHMX000000000
NRZ-37119	NextSeq	7422334	113.7	59	692528	5077694	52	166.2	SAMN17371829	JAFHNX000000000
NRZ-37969	NextSeq	9415590	36.9	178	89885	5183947	52	67.0	SAMN17371833	JAFHOB000000000
NRZ-38718	MiSeq	886134	122.6	435	24473	5068436	52	21.4	SAMN17371837	JAFHOF000000000
NRZ-38954	NextSeq	7102658	36.9	1157	11464	5442038	51	48.1	SAMN17371840	JAFHOI000000000
NRZ-45233	NextSeq	3078852	140.7	93	192447	5503477	52	78.7	SAMN17371858	JAFHPA000000000
sc17238571	NextSeq	5443788	116.6	114	389978	5625113	52	112.8	SAMN17371862	JAFHPE000000000
sc17286987	NextSeq	1918496	128,0	311	56024	5686868	52	43.1	SAMN17371866	JAFHPI000000000
sc18364826	MiSeq	1380044	212.8	86	181678	5078963	52	57.8	SAMN17371879	JAFHPV000000000
sc18407397	NextSeq	18267928	119.2	140	337752	5237847	52	415.7	SAMN17371886	JAFHQC000000000
sc18412584	NextSeq	5163230	134.6	83	487805	5100306	52	136.2	SAMN17371888	JAFHQE000000000
sc18412783	NextSeq	5509672	131.5	83	170962	4997670	52	144.9	SAMN17371889	JAFHQF000000000
sc19444294	NextSeq	7167902	127.6	80	337489	5363202	51	170.5	SAMN17371900	JAFHQQ000000000
sc19446287	NextSeq	3308700	132,0	136	147833	5324169	52	82.0	SAMN17371903	JAFHQT000000000
Survcare036	MiSeq	1442396	184.8	216	77323	5525291	52	48.2	SAMN17371948	JAFHSM000000000
Survcare050	NextSeq	2520620	122.3	196	90740	5476061	52	56.2	SAMN17371962	JAFHTA000000000
Survcare080	NextSeq	3565214	131.6	120	176463	5392833	51	87.0	SAMN17372001	JAFHUN000000000
Survcare083-1	NextSeq	6404240	131.1	58	554586	5397907	52	155.7	SAMN17372004	JAFHUQ000000000
Survcare092	NextSeq	2463614	127.9	124	178037	5433118	51	57.9	SAMN17372014	JAFHVA000000000
Survcare120	NextSeq	3185962	110,0	70	338383	5223944	52	67.0	SAMN17372039	JAFHVZ000000000
Survcare132	NextSeq	7832216	121.2	128	350592	5659887	52	167.7	SAMN17372051	JAFHWL000000000
Survcare137	MiSeq	2345034	175.2	124	138095	5450346	52	75.3	SAMN17372053	JAFHWN000000000
Survcare150	NextSeq	10238604	114.8	120	199532	5614486	52	209.3	SAMN17372063	JAFHWX000000000
Survcare154	NextSeq	1160096	120,0	117	122785	5530281	52	25.1	SAMN17372066	JAFHXA000000000
Survcare160	NextSeq	1423218	116.4	212	136630	5412987	51	30.6	SAMN17372072	JAFHXG000000000
Survcare161	NextSeq	1030790	115.3	146	101906	5210629	52	22.8	SAMN17372073	JAFHXH000000000
Survcare162	NextSeq	917716	115.4	326	34672	5159728	52	20.5	SAMN17372074	JAFHXI000000000
Survcare163	NextSeq	859732	114.4	371	48045	5278687	52	18.6	SAMN17372075	JAFHXJ000000000
Survcare165	NextSeq	24852458	118.2	94	195364	5042749	52	582.5	SAMN17372077	JAFHXL000000000
Survcare188	NextSeq	4361332	135.9	114	242797	5425346	52	109.2	SAMN17372097	JAFHYE000000000
Survcare235	MiSeq	1412266	161.3	117	109131	5083010	52	44.8	SAMN17372126	JAFHZG000000000
Survcare239	MiSeq	4161136	162.1	123	167872	5369257	52	125.6	SAMN17372130	JAFHZK000000000
Survcare242	MiSeq	905690	140.8	113	151324	5153293	52	24.7	SAMN17372133	JAFHZN000000000
Survcare247	NextSeq	4307898	108.7	90	189762	5293350	52	88.4	SAMN17372138	JAFHZS000000000
Survcare252	NextSeq	3717604	121.7	126	156774	5301727	52	85.3	SAMN17372143	JAFHZX000000000
Survcare253	NextSeq	5251570	119,0	168	117002	4971544	50	125.7	SAMN17372992	JAFHEJ000000000
Survcare306	MiSeq	539460	162.3	581	19524	5278427	52	16.5	SAMN17372993	JAFHEK000000000
Survcare311	NextSeq	3324406	110,0	93	279542	5382572	52	67.9	SAMN17372996	JAFHEN000000000
Survcare314	NextSeq	758506	110.4	1013	10802	5587804	52	14.9	SAMN17372999	JAFHEQ000000000
Survcare315	MiSeq	734976	180.9	102	182471	5247961	52	25.3	SAMN17373000	JAFHER000000000
Survcare316	MiSeq	684730	165.4	105	212466	5159069	52	21.9	SAMN17373001	JAFHES000000000
Survcare320	NextSeq	2830270	113.8	108	246078	5278951	52	61.0	SAMN17373003	JAFHEU000000000
Survcare336	NextSeq	3033486	106.8	47	423121	4940785	52	65.5	SAMN17373017	JAFHFI000000000
Survcare347	NextSeq	6452720	123,0	65	425812	4884842	54	162.4	SAMN17373027	JAFHFR000000000
Survcare396	NextSeq	1202112	111.3	213	89634	5384741	52	24.8	SAMN17373064	JAFHHC000000000
Survcare397	NextSeq	1753432	110,0	84	168380	4850364	54	39.7	SAMN17373065	JAFHHD000000000
Survcare405	NextSeq	2511860	127.6	274	54597	5195100	52	61.6	SAMN17373073	JAFHHL000000000
Survcare410	NextSeq	2653664	124.3	111	126305	5137615	52	64.2	SAMN17373078	JAFHHQ000000000
Survcare429	NextSeq	1325928	130.2	117	166880	5289103	52	32.6	SAMN17373092	JAFHIE000000000
Survcare457	NextSeq	7395134	117.8	124	236617	5382929	51	161.8	SAMN17373116	JAFHJB000000000
Survcare462	NextSeq	5890684	116.6	59	281554	4920770	52	139.5	SAMN17373120	JAFHJF000000000
ur18060060	NextSeq	4269056	110.1	145	316593	5523667	52	85.0	SAMN17373131	JAFHJQ000000000
ur19075683	NextSeq	5846848	122.9	137	118396	5102860	52	140.8	SAMN17373134	JAFHJT000000000

Supplementary Table S3. Genomic characteristics of the *Citrobacter spp.* isolates from 2016 to 2019 in Hesse, Germany

Isolate	Species	MLST type	Plasmid Inc type and plasmid MLST typing	Antibiotic resistance genes (ARGs)														Total No. Of ARGs	Alternations of outer membran porine genes	
				Carbapenemase	Additional beta-lactam-resistance genes					Aminoglycoside	Fluoroquinolone	MLS - Macrolide, Lincosamide and Streptogramin B	Phenicol	Rifampicin	Sulphonamide	Tetracycline	Trimethoprim			
					CMY-	CTX-M	OXA-	TEM-	others										ompC (374 aa)	ompF (358 aa)
Survcare050	C. freundii	8	IncFII[F2:A-:B-], IncL/M(pOXA-48), TrfA	OXA-48	79	15	1	1B		aac(3)-IIa, aac(6´)-If, aadA1	aac(6')Ib-cr, qnrS1		catB3		sul1, sul2	tet(A), tet(B)	dfrA1	16	1	wild type
bk18035705	C. freundii	18	IncA/C2, IncR	KPC-3	16, 79		10			aacA4, aadA1, aadA5, aph(3')-Ic, aph(3')-VIb, strA, strB	aac(6')Ib-cr		cmlA1, floR	ARR-2	sul1, sul2	tet(A)	dfrA14, dfrA17	20	>2	wild type
NRZ-45233	C. freundii	18	IncHI1A(CIT),IncHI1B(CIT), IncR, IncFII, IncFIB(pHCM2)	OXA-48	79	1	1	1B	DHA-1	aadA5	aac(6')Ib-cr, qnrB4	mph(A)	catB3	ARR-3	sul1		dfrA17	14	>2	wild type
sc18364826	C. freundii	18	IncA/C2, IncR	KPC-3	16, 79		10			aacA4, aadA1, aadA5, aph(3')-Ic, aph(3')-VIb, strA, strB	aac(6')Ib-cr		cmlA1, floR	ARR-2	sul1, sul2	tet(A)	dfrA14, dfrA17	20	>2	wild type
sc18412584	C. freundii	18	IncA/C2, IncR	KPC-3	16, 79		10			aacA4, aadA1, aadA5, aph(3')-Ic, aph(3')-VIb, strA, strB	aac(6')Ib-cr		cmlA1, floR	ARR-2	sul1, sul2	tet(A)	dfrA14, dfrA17	20	>2	wild type
Survcare120	C. freundii	18	IncFII, IncR	OXA-48	79		1	1B	DHA-1	aadA5	aac(6')Ib-cr, qnrB4	mph(A)	catB3	ARR-3	sul1		dfrA17	14	>2	wild type
Survcare161	C. freundii	18	IncR, IncFII, IncX1, IncFIB(pHCM2), IncF [F2:A-:B-]	-	79		1	1B	DHA-1	aadA5	aac(6')Ib-cr, qnrB4	mph(A)	catB3	ARR-3	sul1		dfrA17	12	>2	wild type
Survcare162	C. freundii	18	IncFII[F2:A-:B-], IncR, IncX1, IncFIB(pHCM2)	OXA-48	79		1	1B		aadA5	aac(6')Ib-cr	mph(A)	catB3	ARR-3	sul1		dfrA17	11	>2	wild type
ur19075683	C. freundii	18	IncA/C2, IncR, ColRNAI	KPC-3	16, 79		10			aacA4, aadA1, aadA5, aph(3')-Ic, aph(3')-VIb, strA, strB	aac(6')Ib-cr		cmlA1, floR	ARR-2	sul1, sul2	tet(A)	dfrA14, dfrA17	20	>2	wild type
Survcare132	C. freundii	19	IncN[pMLST15], IncFII-FI, TrfA	KPC-2	41		1	1B		aadA2, aph(3´)-Ia, aac(3)-IId, strA, strB	aac(6´)Ib-cr, qnrB2	mph(A)	catB3	ARR-3	sul1		dfrA12, dfrA18	17	>2	wild type
Survcare188	C. freundii	19	IncR, IncQ1, IncFIB(K), IncFII(S), ColNRAI	VIM-2	41	3	1		SHV-31	aadA2, aph(3')-Ic, strA, strB	aac(6')Ib-cr	mph(A)	catB3	ARR-3	sul1, sul2	tet(A)	dfrA12	17	>2	wild type
Survcare242	C. freundii	19	ColRNAI	VIM-4	41		10		SHV-12	aac(3)-Ib, aadA1, aadA2, aadB, strA, strB	qnrA1				sul1		dfrA1, dfrA16	14	>2	>2
Survcare247	C. freundii	19	IncL/M(pOXA-48), IncHI2, IncHI2A, TrfA	OXA-48	41			1B		aadA2, aadB, strA, strB	qnrA1		catA1		sul1, sul2		dfrA16	12	>2	wild type
Survcare315	C. freundii	19	IncL/M(pOXA-48), IncQ1, IncHI2	OXA-48	41			1B	SHV-12	aac(6')-IIc, strA, strB		ere(A)	catA1		sul1, sul2		dfrA18	12	>2	wild type
Survcare316	C. freundii	19	IncL/M(pOXA-48), IncQ1, IncFII(SARC14),	OXA-48	41			1B		strA, strB			catA1		sul2			7	>2	wild type
Survcare320	C. freundii	19	IncN[pMLST7-like], TrfA	VIM-1	41					aacA4, aadA1, strA, strB	aac(6´)Ib-cr						dfrA1, dfrA14	9	>2	wild type
Survcare410	C. freundii	19	IncL/M(pOXA-48), IncQ1, IncFII(S), ColRNAI	OXA-48	41	3		110		strA, strB			catA1		sul2			7	>2	>2
NRZ-31836	C. freundii	22	IncN[pMLST15]	KPC-2	48		1	1B		aac(3)-IId, aadA1, strA, strB	aac(6')Ib-cr, qnrB2	mph(A)	catB3	ARR-3	sul1		dfrA18	15	>2	disrupted
NRZ-37969	C. freundii	22	IncN[pMLST15], ColRNAI	KPC-2	48		1	1B		aac(3)-IId, aadA1, strA, strB	aac(6')Ib-cr, qnrB2	mph(A)	catB3	ARR-3	sul1	tet(A)	dfrA1, dfrA18	17	>2	disrupted
NRZ-38954	C. freundii	22	IncN[pMLST15] partial, IncHI2[ST-1], IncNI2A	KPC-2	48	9		1B		aac(3)-IId, aadB, strA, strB,	aac(6')Ib-cr	mph(A)	catB3	ARR-3	sul1		dfrA1, dfrA18	15	>2	>2
sc17286987	C. freundii	22	IncN3, IncR, TrfA, IncFII, pKPC-CAV1321	KPC-2, VIM-4	48					aadA24, aph(3')-VIa,		mph(A)	catA1		sul1, sul2		dfrA1	10	1	>2
Survcare092	C. freundii	22	IncN[pMLST15], IncHI2A	KPC-2	48	9	1	1B		aac(3)-IId,aadA1, aadA2, aadB, strA, strB	aac(6')Ib-cr, qnrB2	mph(A)	catB3	ARR-3	sul1	tet(A)	dfrA1, dfrA18	20	>2	disrupted
Survcare160	C. freundii	22	IncHI2 [ST-1], IncHI2A, IncN [unknown]	-	48	9		1B		aadA1, aadA2, aadB, aac(3)-IId, strA, strB	aac(6')Ib-cr, qnrB2	mph(A)		ARR-3	sul1	tet(A)	dfrA1, dfrA18	17	>2	disrupted
Survcare252	C. freundii	22	IncR, IncHI1B(CIT), IncFIB(pB171), Rep, ColRNAI	OXA-48	48		1			aadA1	aac(6')Ib-cr	mph(A)	catB3	ARR-3	sul1	tet(A)	dfrA1	11	>2	>2
Survcare429	C. freundii	22	IncX3, IncR, IncFIB(pB171), ColRNAI, Col(MGD2)	NDM-5	48		1	1B	DHA-1	aadA1	aac(6')Ib-cr, qnrB4	mph(A)	catB3, floR	ARR-2	sul1, sul2	tet(A)	dfrA1	16	>2	>2
Survcare396	C. freundii	91	IncL/M(pOXA-48), IncA/C2, IncR	OXA-48	65	15		1B		aadA16	aac(6')Ib-cr, qnrB6, qnrB38	mph(A)		ARR-3	sul1			11	wild type	>2
NRZ-29912	C. freundii	98	IncN[pMLST15], ColRNAI	KPC-2	84		1	1B		aac(3)-IId, strA, strB	aac(6')Ib-cr, qnrB2, qnrB38	mph(A)	catB3	ARR-3	sul1		dfrA18	15	2	>2
NRZ-31131	C. freundii	98	IncN[pMLST15], ColRNAI	KPC-2	84		1	1B		aac(3)-IId, aadA1, strA, strB	aac(6')Ib-cr, qnrB2, qnrB38	mph(A)	catB3	ARR-3	sul1		dfrA18	16	2	>2
ur18060060	C. freundii	98	IncN[pMLST15], IncFIA(HI1)	KPC-2	84		1	1B		aac(3)-IId, strA, strB	aac(6')Ib-cr, qnrB2, qnrB38	mph(A)	catB3	ARR-3	sul1		dfrA18	15	2	>2
Survcare306	C. freundii	107	IncN[pMLST15], Col(BS512)	KPC-2	48	15	1			aadB, aac(3)-IIa	aac(6´)Ib-cr, qnrB1	mph(A)	catA1, catB3	ARR-3	sul1			13	>2	disrupted
Survcare405	C. freundii	111	IncN[pMLST15], IncHI1B(CIT)	KPC-2	65		1	1B		aac(3)-IId, strA, strB	aac(6')Ib-cr, qnrB2, qnrB38	mph(A)	catB3	ARR-3	sul1		dfrA18	15	wild type	>2
Survcare239	C. freundii	112	IncL/M(pOXA-48), IncHI2, IncHI2A	OXA-48	75					strA, strB	aac(6')Ib-cr	ere(A)	catA2		sul1		dfrA18	9	>2	wild type
NRZ-38718	C. freundii	114	IncN[pMLST15]	KPC-2	48			1B		aac(3)-IId		mph(A)						5	>2	wild type
Survcare036	C. freundii	116	IncN[pMLST15], IncHI2[ST-1]	KPC-2	65		1	1B	DHA-1	aac(3)-IId, aac(6´)-Iic, strA,strB	aac(6')Ib-cr, qnrB2, qnrB4	mph(A), ere(A)	catB3	ARR-3	sul1		dfrA18	18	>2	wild type
Survcare080	C. freundii	256	IncN[pMLST15]	KPC-2	51		1	1B		aac(3)-IId, strA, strB	aac(6')Ib-cr, qnrB2	mph(A)	catB3	ARR-3	sul1	tet(A)	dfrA18	15	>2	wild type
Survcare457	C. freundii	327	IncN[pMLST15], ColRNAI	KPC-2	48	15	1	1B		aac(6')-If, aac(3)-IId, aac(3)-IIa	aac(6')Ib-cr, qnrB2	mph(A)	catB3	ARR-3	sul1	tet(A)	dfrA18	16	>2	wild type
sc19446287	C. freundii	396	IncFII(S), ColRNAI	VIM-2	78		17			aacA4, aadA1, aadB, aph(3')-Ia	aac(6')Ib-cr				sul1			9	>2	>2
Survcare150	C. freundii	396	IncL/M(pOXA-48), IncHI1A(CIT), IncHI1B(CIT), IncFII(SARC14)	OXA-162	78			2										3	>2	>2
Survcare165	C. freundii	415	IncN[pMLST15], IncFIB(K), ColRNAI	KPC-2, VIM-1	48					aacA4, aadA1, aadA16, aph(3')-Ia, aph(4)-Ia, aac(3)-IVa	aac(6')Ib-cr, qnrB6			ARR-3	sul1		dfrA27	14	>2	wild type
Survcare314	C. freundii	488	IncL/M(pOXA-48), IncN[pMLST6], IncHI1B/A(CIT), ColRNAI	OXA-48	76	3					qnrB38						dfrA14	5	>2	>2
Survcare137	C. freundii	512	IncHI[ST-11], IncFIB(K), IncHI1A(CIT)	NDM-1	65		1	1B		aac(3)-IIa, aadA2, aph(3')-Ia, aph(3')-VIa, strA, strB	aac(6´)Ib-cr	mph(A)	catB3		sul1, sul2	tet(A)	dfrA12	17	>2	wild type
sc19444294-1	C. freundii	540	IncFIB(pNDM-Mar), IncHI1B	NDM-5	79	15				aph(3')-Ia, aph(3')-VIa, armA	qnrS1	mph(A), mph(E), msr(E)	catA1		sul1		dfrA5	13	>2	wild type
NRZ-37119	C. freundii	584	IncN[pMLST7]	VIM-1	79					aacA4	qnrS1					tet(B)	dfrA14	6	>2	wild type
Survcare311	C. freundii	New 1	IncL/M(pOXA-48), TrfA, ColRNAI	OXA-162	75					strB, aac(6')-If					sul2		dfrA1	6	>2	wild type
Survcare163	C. freundii	New 2	IncL/M(pOXA-48), TrfA, IncFII	OXA-48	65	15					qnrB38							4	wild type	>2
sc17238571	C. freundii	New 3	IncHI2 [ST-1], IncFII, IncHI2A, IncFIB(pB171), TrfA, IncF [F2:A-:B70]	VIM-2	51	9	1	1B	SHV-12	aac(3)-IId, aacA4, , aadA2, aadA5, aadB, aph(3')-Ic, strA, strB,	aac(6')Ib-cr, qnrA1	mph(A)	catA1, catB3		sul1		dfrA16, dfrA17	23	>2	>2
Survcare347	C. koseri	NA	IncN[pMLST15], IncI1[ST-36], ColRNAI, IncFII	KPC-2			1	1B	CKO-1	aac(3)-IId, strA, strB	aac(6')Ib-cr, qnrB2	mph(A)	catB3	ARR-3	sul1		dfrA18	14		

Survcare397	*C. koseri*	NA	IncN]pMLST15], ColRNAI	KPC-2		1	1B	MAL_1	*aac(3)-IId, strA, strB*	*aac(6')Ib-cr, qnrB2*	*mph(A)*	*catB3*	*ARR-3*	*sul1*		*dfrA18*	*14*
Survcare336	*C. braakii*	603	IncL/M(pOXA-48)	OXA-48	83												*2*
Survcare462	*C. braakii*	NA		-	74					*qnrB40*							*2*
Survcare083-1	*C. europaeus*	97	IncL/M(pMU407)	VIM-1				CFE-1	*aacA4, aadA1*	*aac(6')Ib-cr, qnrB33*				*sul1*			*7*
Survcare154	*C. europaeus*	New 4	IncFIA(HI1), IncHI1B(R27), IncQ1, IncHI1A, IncHI1[ST-2], IncF[S3*:A8:B-]	VIM-1			1B	CFE-1	*aacA4, aac(3)-IId, aadA2, strA, strB*	*aac(6')Ib-cr, qnrB33*	*mph(A)*			*sul1, sul2*	*tet(B)*	*dfrA12*	*15*
Survcare235	*C. portucalensis*	96	IncFIB(pB171), ColRNAI	VIM-1	46		1B		*aadA1, aacA4, aph(3')-XV, aadA2, aac(3)-IId*	*aac(6')Ib-cr, qnrB57*	*mph(A)*	*catB2*		*sul1, sul2*	*tet(D)*	*dfrA12*	*16*
sc18407397	*C. portucalensis*	232	IncA/C2, IncHI2 [ST-1], IncQ1,	KPC-3	13	1	1B	SHV-12	*aph(3')-Ia, aac(6')-IIc, aac(3)-IId, aph(3')-VIb, strA, strB*	*aac(6')Ib-cr, qnrB18*	*mph(A), ere(A)*	*catA2, floR*		*sul1, sul2*	*tet(A), tet(D)*	*dfrA14, dfrA17*	*23*
Survcare308	*C. portucalensis*	408	IncFII, IncFIB(AP001918), ColRNAI, IncF[F2:A20*:B1]	NDM-5	2	1	1B		*aadA5, aac(3)-IIa*	*aac(6´)Ib-cr, qnrB7*	*mph(A)*	*catB3*		*sul1*	*tet(B)*	*dfrA17*	*14*
sc18412783	*C. portucalensis*	unknown	IncN[pMLST15], ColRNAI	KPC-2	77	1	1B		*aac(3)-IId, strA, strB*	*aac(6')Ib-cr, qnrB2, qnrB42*	*mph(A)*	*catB3*	*ARR-3*	*sul1*		*dfrA18*	*15*
